# Design of Smart Steering Wheel for Unobtrusive Health and Drowsiness Monitoring

**DOI:** 10.3390/s21165285

**Published:** 2021-08-05

**Authors:** Branko Babusiak, Adrian Hajducik, Stefan Medvecky, Michal Lukac, Jaromir Klarak

**Affiliations:** 1 Department of Electromagnetic and Biomedical Engineering, University of Zilina, 01026 Zilina, Slovakia; 2 Department of Design and Machine Elements, University of Zilina, 01026 Zilina, Slovakia; adrian.hajducik@fstroj.uniza.sk (A.H.); michal.lukac@fstroj.uniza.sk (M.L.); 3 Institute of Competitiveness and Innovation, University of Zilina, 01026 Zilina, Slovakia; stefan.medvecky@fstroj.uniza.sk; 4 Department of Automated Production Systems, University of Zilina, 01026 Zilina, Slovakia; jaromir.klarak@fstroj.uniza.sk

**Keywords:** steering wheel, driver monitoring, electrocardiography, photoplethysmography, oximetry, inertial measurement unit

## Abstract

This article describes the design of a smart steering wheel intended for use in unobtrusive health and drowsiness monitoring. The aging population, cardiovascular disease, personalized medicine, and driver fatigue were significant motivations for developing a monitoring platform in cars because people spent much time in cars. The purpose was to create a unique, comprehensive monitoring system for the driver. The crucial parameters in health or drowsiness monitoring, such as heart rate, heart rate variability, and blood oxygenation, are measured by an electrocardiograph and oximeter integrated into the steering wheel. In addition, an inertial unit was integrated into the steering wheel to record and analyze the movement patterns performed by the driver while driving. The developed steering wheel was tested under laboratory and real-life conditions. The measured signals were verified by commercial devices to confirm data correctness and accuracy. The resulting signals show the applicability of the developed platform in further detecting specific cardiovascular diseases (especially atrial fibrillation) and drowsiness.

## 1. Introduction

Civilizational diseases refer to a group of diseases that have spread widely among the human population. These are degenerative diseases that result from the degeneration of tissues and organs. They are most common in developed or third-world countries [1]. In many cases, they are a common cause of death. Civilization diseases are most often divided into cardiovascular diseases, cancer metabolic diseases, and nervous and mental diseases. In addition, the lifestyle of the population has changed significantly over the last 70 years, the pace of life has accelerated, and many people are living under constant stress [2].

Chronic diseases or otherwise non-communicable diseases (NCDs) kill 41 million people each year, representing 71% of all deaths worldwide. Cardiovascular diseases account for the most deaths from so-called non-communicable chronic diseases, at 17.9 million people per year. Tobacco use, physical inactivity, harmful alcohol consumption, and unhealthy diets all increase the risk of death from NCD. The detection, screening, treatment of NCDs, and palliative care are the key components of the NCD response. Early detection and early treatment play the most critical role. There is evidence that such interventions are an excellent economic investment because, if provided to patients promptly, they can reduce the need for more expensive treatment [3]. This fact is confirmed by the current goals of the WHO, which focus on the prevention and overall management of these diseases. In addition, many studies have shown that the risk factors for diseases of the circulatory system and other chronic diseases are shifting to ever-younger age categories [4,5,6,7].

One approach is personalized medicine. It is an approach that exploits the knowledge of the molecular origin of the disease and the knowledge of how the treatment works, in conjunction with the knowledge of the differences between patients [8]. The personalized medicine strategy aims to provide drugs and diagnostic devices that bring patients a tangible improvement in health and quality of life. The critical element is the previously mentioned diagnostic and measuring devices, also called wearables. Wearable technologies, such as medical technologies, become an integral part of personal analysis, physical condition measurement, physiological parameters, or a treatment information plan. They can provide continuous health data on metabolic status, diagnosis, or treatment [9]. They can be conveniently placed on the epidermis, whether introduced through the skin and body gates, to measure electrophysiological or biochemical signals, or to deliver drugs [10]. In some cases, they are built into clothing [11] or other smart elements such as watches [12] or bracelets [13], or are part of various control devices.

Despite the efforts around the world to reduce greenhouse gas emissions produced by cars [14], we can still state that people spend a considerable amount of their life in a car. The time spent in the car is an opportunity to collect physiological information to assess health conditions and sleepiness. According to the European Transport Safety Council, driver fatigue is a significant factor in approximately 20% of commercial road transport crashes [15]. According to surveys, more than half of long-haul drivers have fallen asleep behind the wheel [15]. Sahayadhas [16] observed differences between the alert and drowsy states of the driver in the signal acquired by an electrocardiograph (ECG). Awais [17] has successfully improved drowsiness detection based on a combination of ECG and EEG. The heart rate variability (HRV) calculated from the ECG signal was used as the primary parameter for the drowsiness detection. Lee [18] investigated the robust and distinguishable HRV signals acquired by wearable ECG or photoplethysmograph (PPG) sensors for driver drowsiness detection.

However, the previous studies monitored the ECG using commercial devices or systems that were not permanently built into the car equipment, such as the steering wheel or seat. The ECG electrodes can be inbuilt in cars in different places, but especially in the steering wheel (conductive), driver’s seat (capacitive), or backrest (capacitive—thoracic or lumbar) [19]. The seat-integrated electrocardiography (ECG), capacitive ECG (cECG), and ballistocardiography (BCG) have been applied extensively in the automotive environment. The steering wheel has a decisive advantage because more important signals can be monitored by the driver’s hands making contact with the steering wheel than in the driver’s seat. The study of the current state helped us identify critical parameters for measuring the driver’s cardiovascular health or drowsiness [20,21,22]. This research shows that the steering wheel collecting health describing data is very possible in the research area [17,19,23].

Steering wheel data for drowsiness detection have been the aim of several studies. Studies show that a drowsy driver has abnormal characteristics when operating a steering wheel. For example, the amount of control and operation with the steering wheel decreases, reducing the accuracy and frequency of the rotation of the steering wheel while driving [24]. Li [25] analyzed steering wheel angle (SWA) and yaw angles (YA) at different fatigue conditions. Approximate entropy (ApEn) was used during fatigue detection together with a backpropagation neural network classifier. The experiment lasted 15 h in real traffic, and the data obtained were then marked with a certain degree of driver fatigue. This research achieved 87.21% accuracy in detection. Mortazavi [26] examined several different driver-vehicle control variables, such as SWA, lane-keeping, etc., correlated with drowsiness levels. In addition, several commercial drivers were tested in a simulated environment, and different variables were recorded. The study shows that drowsiness has a significant impact on lane-keeping and steering control behavior. The significance and effectiveness of non-obtrusive drowsiness detection based on parameters such as lateral deviation from the road centerline, lateral acceleration, yaw rate, YA, and steering wheel velocity is highlighted by recent research conducted by Arefnezhad [27]. This data was processed by using deep neural networks; features can be extracted automatically from pre-processed data. The proposed method was based on convolutional neural networks (CNN) and recurrent neural networks (RNN). As a result, the highest accuracy of 96.0% has been achieved with a long short-term memory (LSTM) CNN.

Besides detecting drowsiness, the ECG signal can be used to detect cardiovascular disease (CVD). In Europe, CVD causes 3.9 million deaths and accounts for 45% of all deaths [28]. Atrial fibrillation (AF) is the most common type of cardiac arrhythmia associated with an increased risk of stroke. AF is responsible for approximately one-third of hospitalizations for cardiac rhythm pathologies. It is a serious public health problem due to the higher risk of death and the high financial cost (hospitalization, drug treatment, etc.). [29] AF can be effectively detected by one-lead ECG devices operated by smartphones [30,31,32,33,34]. For instance, the usability of one-lead ECG for the detection of pathological diagnoses was tested in [31]. The atrial fibrillation (AF), atrial flutter, atrioventricular block, regular supraventricular rhythm, and cardiac pacing were defined as pathologies. The one lead ECG device provides a sensitivity of 75% and a specificity of 97% to differentiate the healthy and pathological heart activity. If the abnormalities are limited only to AF, then the sensitivity and specificity were 100% and 94%, respectively. A similar smartphone-dependent, one-lead ECG device was used in [35], where the sensitivity and specificity were 92.8% and 100% for AF, 100% and 100% for atrial flutter, and 56.3% and 100% for pacemaker rhythm.

### Motivation and Aim

Based on the above-mentioned facts, the motivation of this research is the development of a comprehensive driver monitoring system, which will be the basis for further improvement with the simultaneous deployment of ongoing research. Although the paper is not aimed directly at detecting heart disease or drowsiness, the usability and quality of the measured data for this purpose are presented.

This paper introduces a unique platform integrating various sensors monitoring the cardio-vascular system (ECG, PPG, and oximetry). In addition, an inertial measurement unit (IMU) has been integrated into the steering wheel, which can record the movement patterns as SWA or YA performed by the driver while driving. The goal of integrating the IMU unit is to provide additional data to the physiological data, thus increasing the ability of the sensory system to detect the stages of fatigue. Furthermore, the system of sensors is integrated into the unique steering wheel construction developed by the authors. The added value of the presented solution is a system that integrates all sensors into one compact unit. The mechanical and electrical hardware design is described in the Materials and Methods section. The signals measured by the developed steering wheels are shown in the Results and Discussion section. The developed steering wheel was tested under laboratory and real-life conditions. This section also discusses the significance of the measured data in further detecting cardiovascular diseases, especially AF and drowsiness.

## 2. Materials and Methods

This section provides a detailed description of the sensor platform integrated into the steering wheel. The platform is a unique combination of sensors that are independent of car electronics. The system of sensors is encapsulated in a durable polymer case. The designed CAD model of the steering wheel was made using SLS (selective laser sintering) technology. SLS is a well-known 3D printing technology, where polymer-based dust is sintered locally in one layer by the action of a laser. Subsequently, a 3D object is created layer by layer. Polyamide PA12 material was used to produce the steering wheel for its excellent mechanical properties, the precision of detail, and its favorable electrical insulating properties.

### 2.1. Electronic Hardware Design

**Figure 1:**
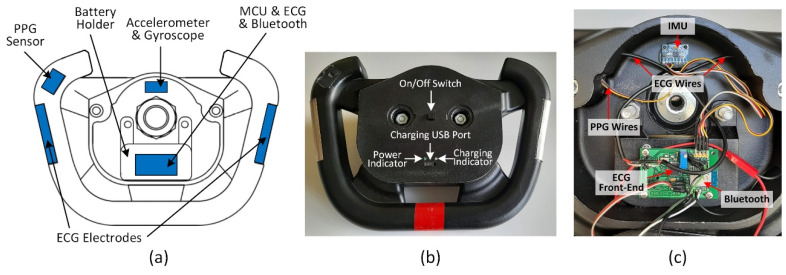
Sketch of smart steering wheel (**a**), real steering wheel design (**b**), and the layout of the electronics inside the steering wheel (**c**).

**Figure 2:**
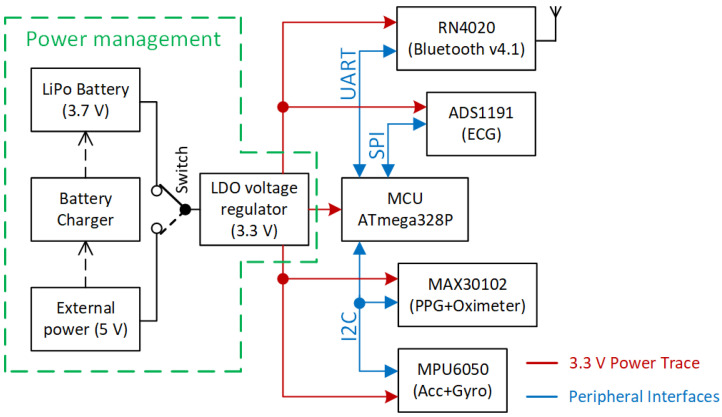
Block diagram of smart steering wheel electronics.

The placement of electronics, components, and cables distribution inside the steering wheel is shown in Figure 1c. The lithium-polymer battery with a capacity of 4000 mAh is placed under the battery holder. The block diagram describing the electronic design of the smart steering wheel (SSW) is depicted in Figure 2. The entire system is powered by a lithium polymer battery with a nominal voltage of 3.7 V. The battery voltage is stepped down to 3.3 V for powering all sensors, microcontroller unit (MCU), and the Bluetooth module. The blocks shown in Figure 2 are described in detail in the following subsections.

#### 2.1.1. ECG Sensor

Electrocardiography is the most used diagnostic method for cardiovascular disease. It is a valuable tool in the detection of any kind of heart abnormality at an early stage. The early diagnosis of heart disease can prevent unnecessary deaths. Therefore, the ECG sensor is an essential part of the steering wheel. In clinical practice, the ECG system with twelve leads is the gold standard. The electrodes placed on the limbs (four electrodes) and the chest (six electrodes) form so-called limb leads and chest leads, respectively. The electrode placed on the right leg provides the return path for common-noise reduction. This type of noise reduction is known as driven-right-leg (DRL) circuit [36,37]. Alternatively, the right leg electrode can be connected to the signal ground to reduce noise from the surroundings. The SSW measures only one channel of ECG from the upper limbs, called lead-I. The usability of one-lead ECG for the detection of AF was already shown in the Introduction section. 

Generally, the conductive gel is placed between the electrode and the skin to reduce electrical impedance between them, resulting in a high-quality ECG signal with a high signal-to-noise ratio (SNR). The ECG system integrated into the steering wheel must deal with high impedance on a skin-electrode interface because of missing the conductive gel [38,39]. Furthermore, the ECG incorporated into the wheel uses only two electrodes without a third electrode, which effectively removes the noise. Removing the third electrode is challenging due to the significantly higher electromagnetic interference (EMI), and lower signal-to-noise ratio (SNR) compared to three-electrode ECG [37,40].

We decided to use the ADS1191 analog front-end (Texas Instruments, Dallas, TX, USA) for the ECG measurement, a part of the ADS1x9x family (Texas Instruments, Dallas, TX, USA). This family is a powerful tool in electrical biopotential measurements such as ECG, EMG, and EEG. The ADS1191 enables the digitalization of one input channel by using a 16-bit delta-sigma AD converter. The essential parameters of the ADS1191 are summarized in Table 1.

**Figure 3:**
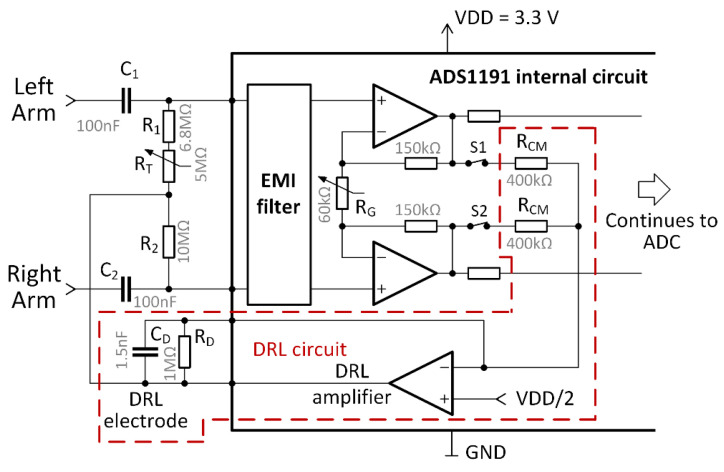
The electrical circuit of two-electrode ECG.

The ADS1191 includes a built-in DRL amplifier and a programable gain amplifier (PGA) used in our design. The voltage of 3.3 V is used for powering the analog and digital parts of the ADS1191. The electrical circuit for the two-electrode ECG is shown in Figure 3.

Figure 3 includes part of an internal circuit of the ADS1191. The value of the internal R_G_ resistor can be digitally tuned to change the differential signal gain and forms the PGA. Six programmable gain values are available: 1, 2, 3, 4, 6, 8, or 12. The DRL circuit can be enabled or disabled by the digitally controlled switches, S1 and S2. The gain and cut-off frequency of the DRL circuit are determined by the values of the external R_D_ resistor and C_D_ capacitor. The *G* gain of the DRL amplifier is computed as [41]:

(1) $$G=-2\cdot \frac{\frac{R_{D}}{1+j\omega R_{D}C_{D}}}{R_{\mathrm{CM}}}=\left(-2\cdot \frac{R_{D}}{R_{\mathrm{CM}}}\right)\cdot \frac{1}{1+j\omega R_{D}C_{D}}=A\cdot \frac{1}{1+j\omega R_{D}C_{D}},$$

where *A* stands for overall DRL gain in the passband. When using values from Figure 3, the gain *A* = −5. The cut-off frequency of the low-pass filter formed by *R*_D_ and *C*_D_ is approx. 106 Hz, and it is computed by

(2) $$f_{c}=\frac{1}{2\pi R_{D}C_{D}}$$

In three-electrode ECG systems, the node denoted as the DRL electrode in Figure 3 is connected to the right leg, or another place on the body, to remove common-mode noise. Our solution is based on a two-electrode ECG presented in [42]. The functionality of this circuit was successfully demonstrated in [37]. The output of the DRL circuit is connected to the midpoint of the resistors (see Figure 3). The *R*_T_ mechanical potentiometer is used to adjust the resistance of the *R*_1_ and *R*_T_ combination to be equal to the *R*_2_ resistance. The output of the DRL circuit biases the ECG inputs (left arm and right arm) through 10 MΩ resistors. The high value of the resistors ensures a high differential mode input impedance, which equals *R*_1_ + *R*_T_ + *R*_2_. Moreover, high resistance values limit the current flowing from the DRL’s output to the subject (driver). The combination of *C*_1_ or *C*_2_ with 10 MΩ resistors provides differential ac-coupling of the ECG signal [42,43]. This ac-coupling is formed by a high-pass filter with the cut-off frequency:

(3) $$f_{c}=\frac{1}{2\pi C_{1}\left(R_{1}+R_{T}\right)}=\frac{1}{2\pi C_{2}R_{2}}=0.16\;\mathrm{Hz}$$

This filter removes the direct current (DC) voltage offset and attenuates the slow fluctuation (below 0.16 Hz) of the ECG isoline.

#### 2.1.2. PPG and Pulse Oximeter Sensor

The MAX30102 (Maxim Integrated, San Jose, CA, USA) is a highly-sensitive pulse oximeter for wearable health monitoring. It includes red and infrared (IR) LEDs with a typical wavelength of 660 nm and 880 nm, respectively. Each LED is a light source for the measurement of the PPG curve by one photodiode. The internal delta-sigma analog-to-digital converter (ADC) with the 18-bit resolution is used for PPG curve digitalization. The sampling rate can be programmed from 50 samples per second (SPS) to 3200 SPS. The analysis of the PPG curve can determine the heart rate [44], blood vessel elasticity [45,46], or cuffless blood pressure [47]. The light absorption at various wavelengths (660 nm and 880 nm) differs between oxygenated and deoxygenated hemoglobin. The LEDs emit light in cycles when only one LED is activated at a time. Four different pulse widths (LED on time) are available in MAX30102: 69 μs, 118 μs, 215 μs, and 411 μs. The intensity of the LED light is determined by the current flowing through the diodes. The LED current can be programmed from 0 mA to 50 mA to control LED light intensity. All settings of the MAX30102 are summarized in Table 2.

Oxygenated hemoglobin absorbs more infrared light in contrast with deoxygenated hemoglobin, which absorbs more red light. Both PPG curves are used to calculate peripheral blood oxygen saturation level (SpO_2_) [48,49,50,51]. The SpO_2_ is a percentage of oxygen saturated hemoglobin relative to the total blood hemoglobin. Normal SpO_2_ saturation is typically between 95% and 100% for patients without pulmonary pathology. The research [52] shows that SpO_2_ decreases during drowsiness and increases when drowsiness declines. It is thus suitable to monitor the SpO_2_ parameter to avoid car accidents caused by driver fatigue.

The SpO_2_ is calculated using a formula provided by the MAX30102 manufacturer, which was also used in [50]:

(4) $${\mathrm{SpO}}_{2}=-45.060\cdot R^{2}+30.534\cdot R+94.845$$

where *R* is known as the “ratio of ratios”, and the following formula calculates it:

(5) $$R=\frac{\frac{AC_{\mathrm{RED}}}{DC_{\mathrm{RED}}}}{\frac{AC_{\mathrm{IR}}}{DC_{\mathrm{IR}}}}$$

The *DC*_RED_ and *DC*_IR_ are DC components (offsets) of the signals collected from red and infrared LED, respectively. The DC component represents the absorption of the light in the tissue, venous, capillary, bones, etc. The *AC*_RED_ and *AC*_IR_ alternating current (AC) components reflect red and infrared light absorption in the arterial blood, respectively. The calculation of the SpO_2_ is shown on the actual SSW data in the Results and Discussion section.

#### 2.1.3. IMU Sensor

The accelerometer and gyroscope are parts of the inertial measurement unit (IMU). The MPU-6050 (Invensense, San José, CA, USA) is used as the IMU. It is a 3-axis gyroscope and 3-axis accelerometer with an integrated I2C bus. The 16-bit ADCs are used for each gyroscope and accelerometer axis. The IMU has a full-scale programmable range of ±250°/s, ±500°/s, ±1000°/s, and ±2000°/s, and ±2 g, ±4 g, ±8 g, and ±16 g for the gyroscope and accelerometer, respectively. Under normal conditions, the steering wheel does not turn rapidly, so the lowest ranges are used for the gyroscope and accelerometer. The MPU-6050 can be supplied by voltage in the range of 2.375 V to 3.46 V.

The gyroscope and accelerometer data are used to detect motion artifacts in ECG and PPG signals due to steering wheel rotation and road unevenness, such as road potholes and road bumps. Moreover, the IMU data can be used for driver fatigue detection by monitoring steering wheel movements [53,54] or recognizing driving style [55].

#### 2.1.4. Wireless Communication Module

The steering wheel prototype is intended to be used with another device such as a notebook, tablet, or smartphone, which acts as a data processing and visualization unit. The data are transferred wirelessly via Bluetooth version 4.1 by the RN4020 module (Microchip Technology, Chandler, AZ, USA). This version of Bluetooth, also known as Bluetooth Low Energy (BLE), is focused on wireless data transfer for short distances (up to 10 m) in healthcare. The RN4020 provides a maximum data transfer rate of 1 Mbit with low current consumption suitable for battery-powered devices. As shown in [56], the current consumption of RN4020 and the MCU (ATmega328P, Microchip Technology, Chandler, AZ, USA) is only 7.8 mA when using the data rate of 1000 SPS. The Microchip Low-energy Data Profile (MLDP) is used for custom data transfer. The MLDP is a private BLE service that provides a 20 kbps serial data transport over Bluetooth, which is a sufficient data rate for our purpose.

#### 2.1.5. Power Management

**Figure 4:**
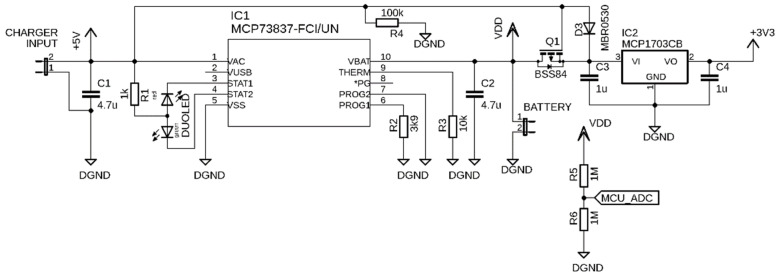
Electric scheme of steering wheel power management.

The powering of the SSW is independent of the car power supply and electronics. The separation of power supplies helps to protect the car electronics from damage while testing the steering wheel prototype. As is shown in Figure 2, the SSW is powered from a lithium-polymer (LiPol) battery with a nominal voltage of 3.7 V. The battery voltage is regulated to 3.3 V by a MCP1703 (Microchip Technology, Chandler, AZ, USA) low-dropout voltage regulator (see schematic in Figure 4), which can deliver a current of 250 mA. The voltage of 3.3 V powers all sensors of the SSW. The battery voltage is monitored by the ADC of the MCU via a voltage divider formed by resistors R5 and R6 in Figure 4. The battery can be charged by an external power source such as a car USB charger or power bank. The micro-USB connector is used as charger input. The MCP73837 (Microchip Technology, Chandler, AZ, USA) standalone battery charge management controller plays an essential part in power management. It charges the battery by a current determined by the R2 resistor. The charging current is 256 mA when the value of 3.9 kΩ is used. A bicolor LED indicates the charging process on the SSW front panel. The red light and green light indicate the charging process and charging completed status, respectively. During charging, the electronics of SSW are not powering by the battery but by an external power source. This feature is provided by P-channel MOSFET BSS84 (Q1), which acts as an electronic switch. The battery disconnection ensures proper battery charging while the system is externally powered.

### 2.2. Data Acquisition

**Figure 5:**
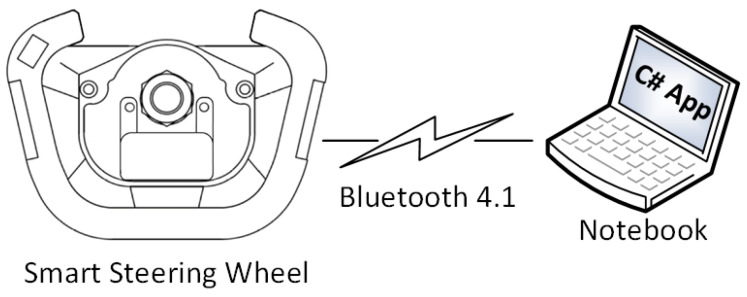
Testing scenario using smart steering wheel and notebook.

The smart steering wheel is connectable via Bluetooth to any device running a dedicated application. For the functionality test, the application in the C# programming language was developed. The application runs on a notebook running Windows 10. The testing scenario is depicted in Figure 5. In practice, the notebook can be replaced by a smartphone, tablet, or car display running Android Auto.

The smart steering wheel can work in four operating modes, which are summarized in Table 3. The notebook application provides the mode selection and the setting of custom signal acquisition parameters. The acquired data are real-time plotted in all available modes.

If the ECG, PPG, or IMU mode is activated, the other unused sensors are in sleep mode to reduce the power consumption. The IMU mode provides the three-axis accelerometric and gyroscopic data, and is advantageous in detecting driver fatigue or recognition of driving style.

**Figure 6:**
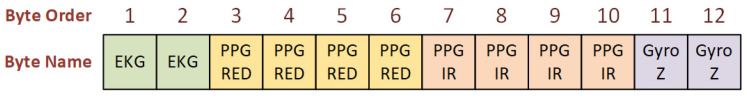
COMBO mode data frame.

The COMBO mode (Figure 6) provides complex information from all sensors. The IMU data are reduced to gyroscopic data in the Z-axis (Gyro Z) because this data best represents steering wheel movements. The suitability of Gyro Z data selection is discussed in the next section.

## 3. Results and Discussion

**Figure 7:**
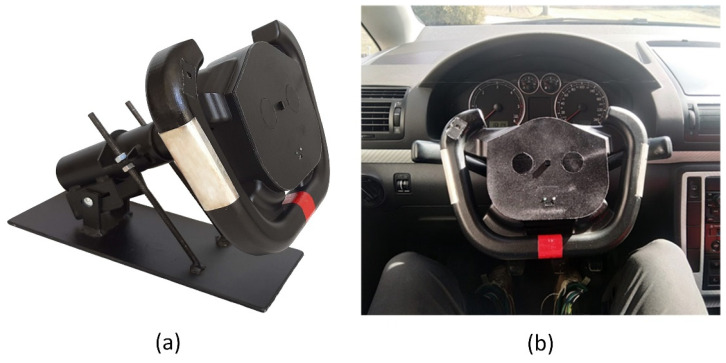
Two testing options—The SSW mounted on the console (**a**) and in the car (**b**).

A series of experiments were conducted to verify the functionality of the SSW and the correctness of the measured data. This section provides measurements of signals from all sensors integrated into the SSW. The signals measured from the SSW are compared to signals acquired from accurate commercial devices. The SSW was tested in the laboratory and real-life conditions. The unique console was constructed for the SSW fixation (Figure 7a) in the laboratory tests. The inclination of the SSW mounted to the console is adjustable, so it is possible to set the same steering wheel inclination as in the car. The SSW was mounted into the car (a Volkswagen Sharan) to provide preliminary tests in real-life conditions during driving (Figure 7b). SSW is compatible with the VW Group for most models of the Škoda, Seat, and Audi. During the tests, the steering wheel airbag was not used, and the car was driving on roads without the traffic to ensure the driver’s safety. The measured data are visualized and analyzed in MATLAB R2018b (Mathworks, Inc., Natick, MA, USA).

### 3.1. Measurement of ECG Signal

**Figure 8:**
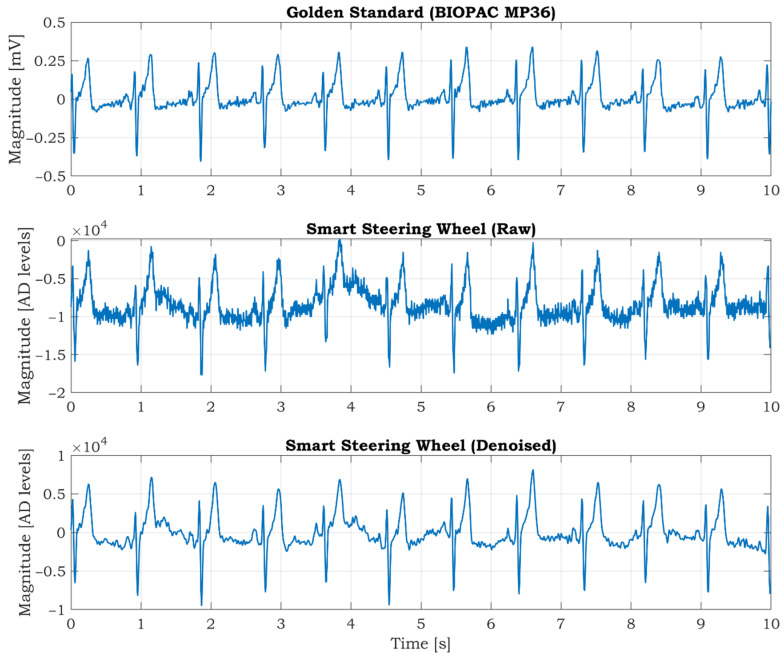
The synchronized ECG signal measured by BIOPAC MP36 (top) and developed smart steering wheel (middle) in the laboratory environment. The filtered ECG signal from the smart steering wheel (bottom). Filtration is based on wavelets.

The BIOPAC MP36 acquisition unit (BIOPAC Systems Inc., Goleta, CA, USA) is used as a commercial high precision device to verify the quality of the ECG signals. It comprises precise built-in universal amplifiers and a 24-bit AD converter. The MP36 unit is designed to measure a wide spectrum of physiological signals such as ECG, electromyography (EMG), electroencephalography (EEG), or PPG. The solid gel disposable electrodes are placed on both wrists and the right ankle. The electrode on the ankle acts as a grounding electrode used to suppress noise from the surroundings (mostly mains noise). The ECG signal measured by MP36 is considered a gold standard. The sampling frequency of the MP36 is set to 1000 SPS, and a hardware band-pass filter in the frequency range (0.5–35) Hz is activated. The ECG is synchronously measured by the SSW when the hands are placed on electrode locations, and the subject is calm and relaxed (see Figure 8). The ECG is sampled at 125 SPS by the SSW. The measured signals are shown in Figure 8. As seen from the figure, the signal from the SSW contains more noise, and the ECG isoline fluctuation is more evident than the ECG measured by BIOPAC. The wavelet filtration is applied to the raw signal to remove mentioned undesirable artifacts. The five levels wavelet decomposition using symlet 4 mother wavelet was applied to the raw signal. The denoised signal is recomposed after removing noisy parts from details in levels 3, 4, and 5. The signal offset is removed by using a digital high-pass filter with a 0.5 Hz cut-off frequency. The resulting denoised signal is shown at the bottom of Figure 8. The signal quality is very similar to the gold standard signal measured by the BIOPAC MP36 (top graph in Figure 8).

### 3.2. Measurement of PPG Signal

**Figure 9:**
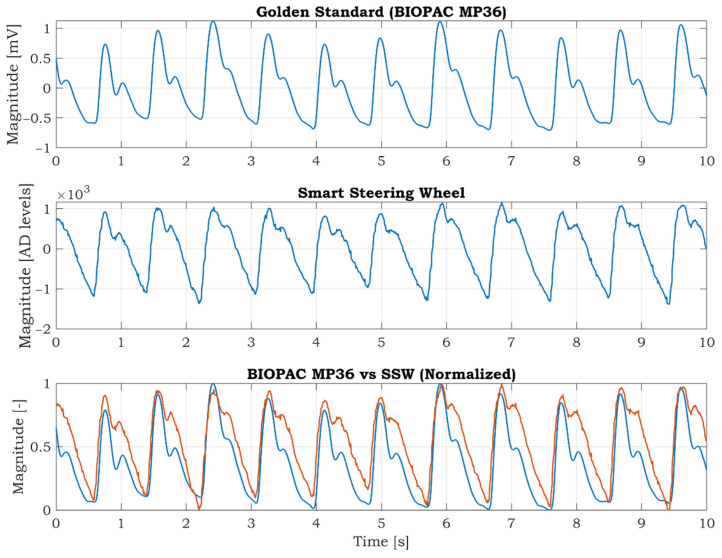
The synchronized PPG signal measured by BIOPAC MP36 (top) and developed smart steering wheel (middle) in the laboratory environment. The comparison between normalized PPG signal measured by BIOPAC (blue) and SSW (red) in the bottom graph.

For the reference measurement of the PPG signal, the BIOPAC MP36 device with the SS4LA PPG transducer is used. The transducer consists of a matched infrared emitter and photodiode detector, and it uses the same reflective principle as the pulse oximeter sensor integrated in the SSW. The SS4LA sensor is placed on the right thumb. The sampling frequency is set to 1000 SPS, and a low-pass filter with a cut-off frequency at 40 Hz is activated. The left thumb is placed on the oximeter sensor of the SSW. The signal from the IR LED is used for comparison; the signal from the RED LED is redundant in this case. The PPG signal of the SSW is sampled at 125 SPS. The synchronized PPG signals from the BIOPAC MP36 and SSW are shown in Figure 9. At first sight, there are differences between the curves. Many factors cause the differences. Firstly, the BIOPAC PPG is fixed to the thumb by a stretchable strap, whereas in the case of the SSW, the thumb is placed freely on the sensor. The unfixed finger produces more artifacts in the signal, so the quality is lower than in the BIOPAC PPG. Furthermore, the PPG signal measured on fingers on different hands can vary because the blood vessel system is not symmetric and depends on the subject’s health condition. Despite these facts, we can conclude that the positions of the peaks are almost the same, as seen in the bottom graph of Figure 9. The positions of the PPG peaks can be used to calculate the BPM or pulse wave velocity if the ECG signal is known.

### 3.3. Measurement of IMU Data

**Figure 10:**
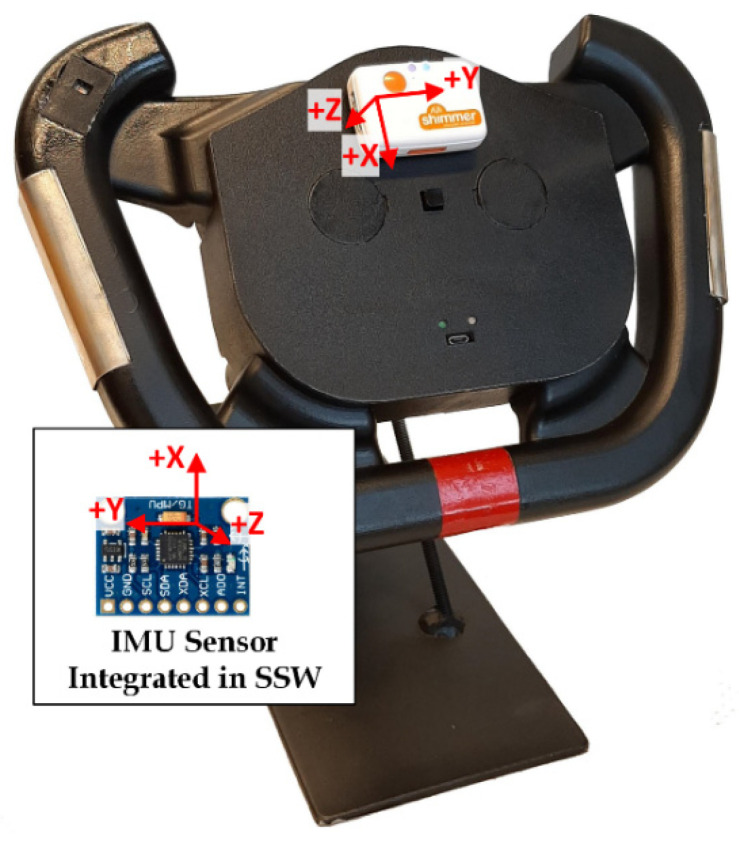
The SSW with Shimmer IMU unit.

The Shimmer3 IMU unit (Shimmer Research Ltd., Dublin, Ireland) is used as a commercial device that provides superior data quality. This unit integrates nine degrees of freedom sensing via accelerometer, gyroscope, and magnetometer. It is placed on the top cover of the SSW (Figure 10) in parallel with the integrated IMU sensor. When comparing the accelerometer axes orientation of the Shimmer and SSW IMU, the X and Y axes are in the opposite direction, and Z-axis has the same direction.

**Figure 11:**
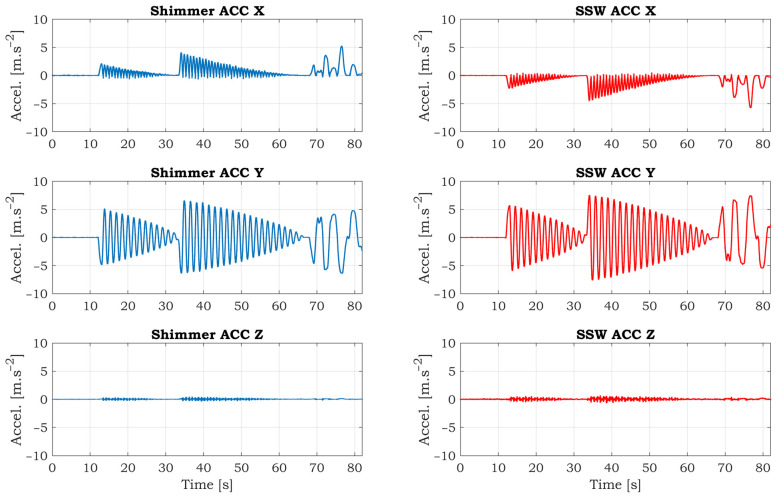
Comparison of accelerometric data measured by reference Shimmer IMU unit (left side) and smart steering wheel (right side) measured in the laboratory environment.

**Figure 12:**
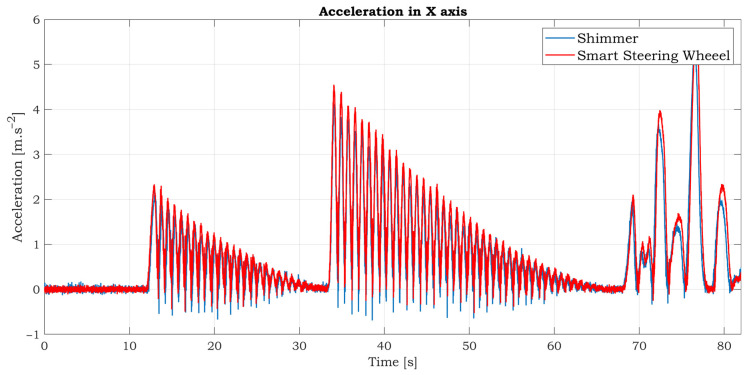
Detailed comparison of accelerometric data in X-axis.

A simple experiment was conducted to verify the accuracy of the accelerometric and gyroscopic data of the SSW. The sampling rate was set to 125 SPS for both devices. The ranges for the accelerometer and gyroscope are also identical. The full-scale range is ±2 g and 250°/s for the accelerometer and gyroscope, respectively. The SSW fixed to the stand has one balanced state shown in Figure 10. When the SSW is rotated by some angle, the SSW tends back to the balanced state. The experiment scenario is as follows: the SSW is grabbed by a hand and rotated by some angle and then dropped. After that, the SSW started to swing until it reached a balanced state. Then the process is repeated with a bigger yaw. After that, a few free clockwise and counterclockwise rotations were performed. The synchronized accelerometric data in the three axes are shown in Figure 11. As can be seen from the graphs, the Shimmer and SSW data are very similar. The data in the X and Y axis are inverted because of the opposite orientations of these axes. The swings of the SSW are almost invisible in the Z-axis because this axis is consistent with the axis of rotation. The detailed comparison of comparable accelerometric data in the X-axis is shown in Figure 12. There are tiny differences between the curves caused by slightly different inclinations between IMUs.

**Figure 13:**
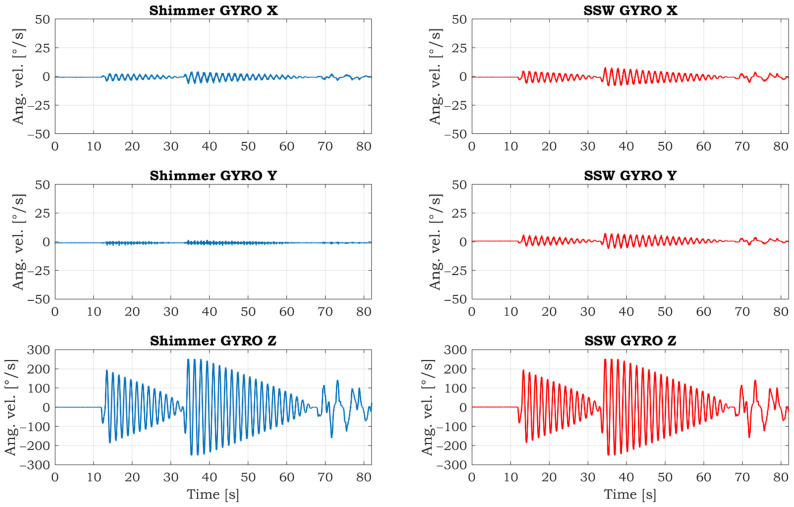
Comparison of gyroscopic data measured by reference Shimmer IMU unit (left side) and smart steering wheel (right side) measured in the laboratory environment.

**Figure 14:**
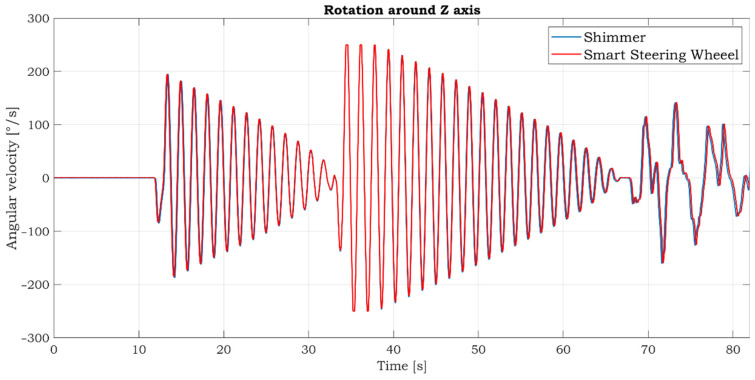
Detailed comparison of gyroscopic data in Z-axis.

The comparative gyroscopic data are shown in Figure 13. As expected, the strongest signal is measured on the Z-axis, which reflects rotation around the SSW’s axis. The details of measured signals in this axis are shown in Figure 14. The data’s high similarity confirms the correctness of the IMU sensor settings and positioning within the inner space of the SSW.

### 3.4. Testing of the SSW in the Car

**Figure 15:**
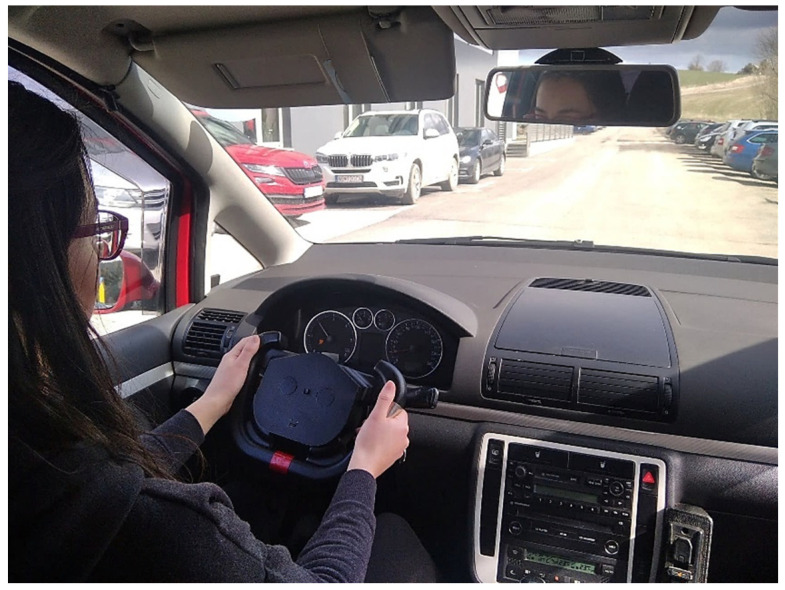
Testing of the SSW while driving.

The SSW was mounted into the car to provide a preliminary test of the functionality in real-life conditions. The test was performed on side roads without traffic to ensure the driver’s safety. The original front airbag is not used because there is not enough space for it in the SSW prototype. The testing scenario is depicted in Figure 15. The long-term use of the SSW in its present form is slightly uncomfortable due to the smaller radius of the SSW when compared to the original steering wheel radius. The next generation of the SSW will have a bigger radius and inner space for the front airbag.

**Figure 16:**
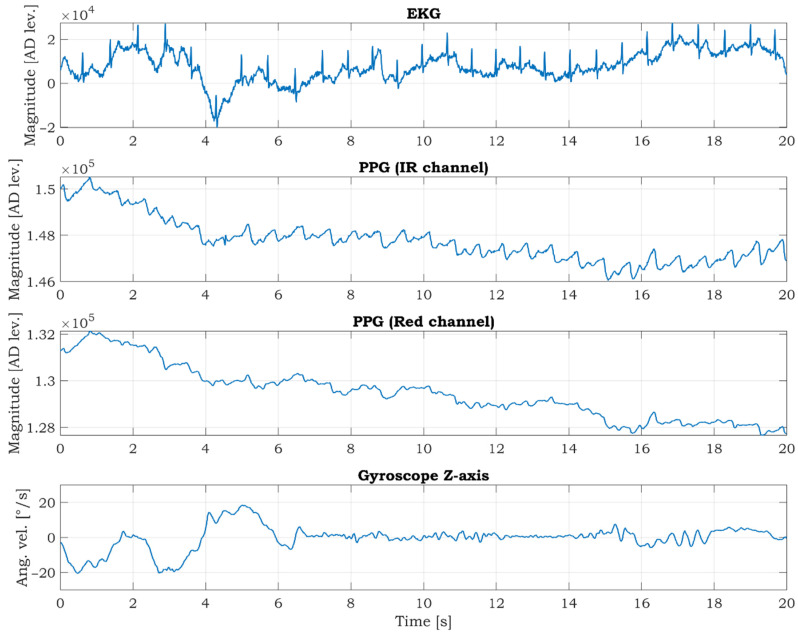
The data acquired in COMBO mode during the driving. The interval of 20 s is shown.

The COMBO mode (described in Section 2.2) was activated during the driving. The representative twenty seconds long segment of acquired signals is shown in Figure 16. The fluctuation of ECG and PPG signals is visible when the SSW is rotated more rapidly in the first 6 s.

The current consumption in the COMBO mode is about 36.5 mA. If we consider a used battery with a capacity of 4000 mAh, the SSW can measure and transfer EEG, PPG, and gyro data continuously over Bluetooth for approx. 109.5 h (4.5 days).

**Figure 17:**
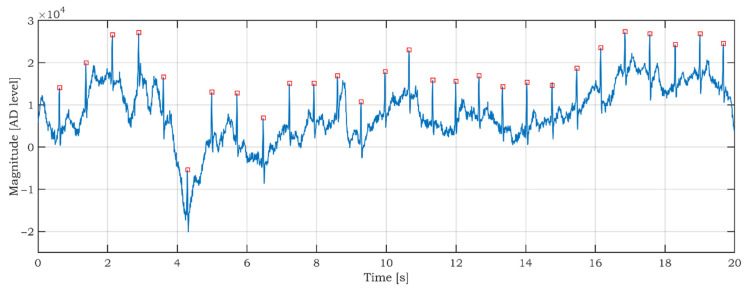
Detection of R peaks in the raw ECG signal measured by the SSW.

Although a detailed data analysis is not the aim of this article, the preliminary data analysis of the car data is done to prove the SSW data’s usability and significance. As seen in Figure 16, the ECG signal is noisy and fluctuating. We applied a modified Pan-Tompkins algorithm for R peaks detection, which was described in [57]. The correct detection of R peaks is a good indicator of ECG signal quality [58,59]. The result of the R peaks detection is shown in Figure 17. The red squares mark the R peaks. As can be seen in Figure 17, all R peaks are detected successfully. Correct R peak detection is crucial for calculating heartbeats per minute (BPM) and heart rate variability (HRV).

**Figure 18:**
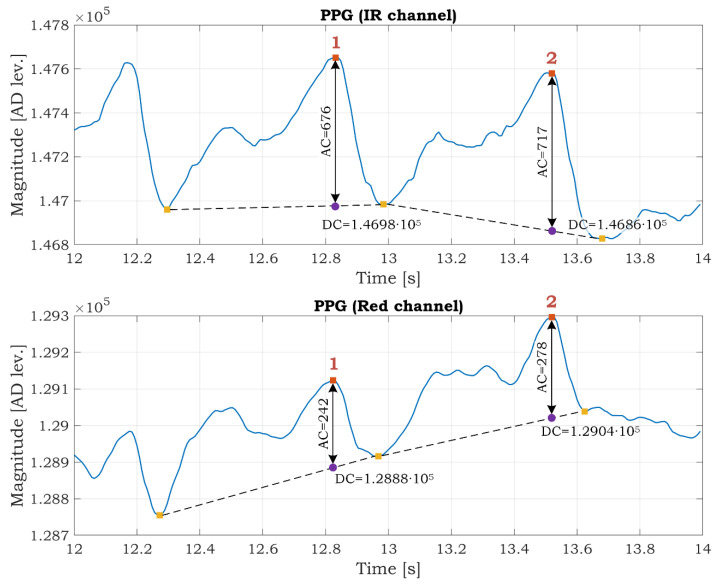
PPG signal in the infrared and red channel with determined AC and DC components.

The PPG signal in the infrared channel has better quality than in the red channel (Figure 16) because red light penetrates to a lesser depth than infrared and carries less information about the arterial blood. The usability of PPG data for the estimation of SpO_2_ is shown in Figure 18. We select a time interval from 12 s to 14 s. We utilize this interval because the SSW was not rotating (see gyroscopic data in Figure 16), so it is assumed that a small portion of movement artifacts are in the signals caused by the rotation of the SSW. There are two peaks marked as 1 and 2 in both channels in Figure 18. The AC and DC components are determined from the graphs. These values are used for the calculation of the *R* ratio according to formula (5). Then the SpO_2_ parameter for both peaks is computed using Equation (4). The SpO_2_ is 99.73% and 99.47% for the first and second peaks, respectively. The values are within the typical range of the SpO_2_. This example demonstrates the possibility of monitoring the SpO_2_ while driving. The SpO_2_ is often determined as the average value in a predefined time interval (several PPG peaks).

The motion artifacts (MA) are a serious problem in PPG measurement and successive SpO_2_ estimation. The MA are caused by every mutual movement between the skin and the PPG sensor, accompanied by changes in optical coupling between the illuminated tissue and the sensor [60]. The research [61] describes the effects of motion on the SpO_2_ readings and summarizes generally used solutions for MA reduction. Averaging the noisy data over a longer time and holding data until clean data are qualified are commonly used as MA reduction methods. The authors in [62] proposed motion artifact reduction based on continuous wavelet transform in PPG signal acquired by a wrist-worn device. In our case, the MA issue in the PPG signal is more critical because the PPG sensor is not fixed to the finger. To increase the reliability of the SpO_2_ estimation, it is highly advisable to reduce MA in the PPG signals. This can be achieved by applying some MA reduction methods described above and by removing high noisy signal segments during rapid SSW movements. Gyroscopic data can effortlessly identify the noisy segments. This fact proves the importance of adding gyroscope data to the COMBO mode.

## 4. Conclusions

The unique design of SSW is presented in this paper. The steering wheel can be used in applications that monitor the driver’s health, drowsiness, or driving style. Alternatively, it can be used to map road irregularities when using additional GPS information.

The reason we focused on the ECG and PPG signals is that these signals start to change in stages that forego the state of drowsiness and fatigue. The camera systems respond to the situation and do not provide the ability to monitor gradual physiological changes. Although the aim of this article was not a detailed analysis of the data, the significance and usability of the data from the SSW were demonstrated by using the Pan-Tompkins algorithm for detecting R peaks from the ECG signal, and by calculating blood oxygen saturation from the PPG signals. The significance and applicability of the health data were also confirmed when performing experimental measurements.

We are currently continuing our measurements. The quality of the measured signals presented in the results gives us a promising motivation to continue in this research, focusing directly on detecting drowsiness and specific types of heart disease (especially AF) using the presented platform. Collecting data from several SSWs to form a database of personalized data that would provide more accurate training data for drowsiness and CVD detection. Personalized data are necessary to create a so-called generalized model while implementing artificial intelligence. Such an approach would significantly advance the issue of driver fatigue detection.

In conclusion, we can state that the presented prototype addresses highly current issues, corresponds to trends and challenges for the future, and its solution actively contributes to progress in research areas such as non-invasive driver monitoring, the digitalization of healthcare, personalized medicine, and telemedicine.

## Figures and Tables

**Table 1:** Most essential parameters of ADS1191.

Parameter/Feature	Value
Resolution	16 bits
Maximal Sampling Rate	8 kSPS
Power Supply (Analog)	2.7 V to 5.25 V
Power Supply (Digital)	1.7 V to 3.6 V
Communication Interface	SPI
Power Consumption (Typ)	335 μW
Input Bias Current	1 nA
CMRR	−95 dB
SNR	92 dB

**Table 2:** Parameters of the pulse oximeter (MAX30102) used in the SSW.

Parameter/Feature	Value
Resolution	18 bits
Sampling Rate	200 SPS
LEDs Current	50 mA
LED Pulse Width	411 μs
Power Supply	3.3 V
Communication Interface	I2C

**Table 3:** The smart steering wheel modes.

Mode	Channels	Sampling Rate [SPS]	Bits/Sample
ECG	1	125 or 500	16
PPG	2	200	18
IMU	6	125	16
COMBO	4	125	mix

## Data Availability

The data presented in this study are available on request from the corresponding author.

## References

[1] Kopp W. (2019). How Western diet and lifestyle drive the pandemic of obesity and civilization diseases. Diabetes Metab. Syndr. Obes. Targers Ther..

[2] Chrousos G.P. (2009). Stress and disorders of the stress system. Nat. Rev. Endocrinol..

[3] Forouzanfar M.H., Afshin A., Alexander L.T., Biryukov S., Brauer M., Cercy K., Charlson F.J., Cohen A.J., Dandona L., Estep K. (2016). Global, regional, and national comparative risk assessment of 79 behavioural, environmental and occupational, and metabolic risks or clusters of risks, 1990–2015: A systematic analysis for the Global Burden of Disease Study 2015. Lancet.

[4] Timmis A., Townsend N., Gale C., Grobbee R., Maniadakis N., Flather M., Wilkins E., Wright L., Vos R., Bax J. (2018). European Society of Cardiology: Cardiovascular disease statistics 2017. Eur. Heart J..

[5] Leatherdale S.T. (2015). An examination of the co-occurrence of modifiable risk factors associated with chronic disease among youth in the COMPASS study. Cancer Causes Control.

[6] Lucan S.C. (2015). Concerning limitations of food-environment research: A narrative review and commentary framed around obesity and diet-related diseases in youth. J. Acad. Nutr. Diet..

[7] Li Y., Lawley M.A., Siscovick D.S., Zhang D., Pagán J.A. (2016). Agent-based modeling of chronic diseases: A narrative review and future research directions. Prev. Chronic Dis..

[8] Schork N.J. (2015). Personalized medicine: Time for one-person trials. Nature.

[9] Yetisen A.K., Martinez-Hurtado J.L., Ünal B., Khademhosseini A., Butt H. (2018). Wearables in medicine. Adv. Mater..

[10] Dunn J., Runge R., Snyder M. (2018). Wearables and the medical revolution. Pers. Med..

[11] Chen M., Ma Y., Song J., Lai C.F., Hu B. (2016). Smart clothing: Connecting human with clouds and big data for sustainable health monitoring. Mob. Netw. Appl..

[12] Chan M., Estève D., Fourniols J.Y., Escriba C., Campo E. (2012). Smart wearable systems: Current status and future challenges. Artif. Intell. Med..

[13] Athavale Y., Krishnan S. (2017). Biosignal monitoring using wearables: Observations and opportunities. Biomed. Signal Process. Control.

[14] Šarkan B., Kuranc A., Kučera L. (2019). Calculations of exhaust emissions produced by vehicle with petrol engine in urban area. IOP Conf. Ser. Mater. Sci. Eng..

[15] European Transport Safety Council (2001). The Role of Driver Fatigue in Commercial Road Transport Crashes.

[16] Sahayadhas A., Sundaraj K., Murugappan M. (2013). Drowsiness detection during different times of day using multiple features. Australas. Phys. Eng. Sci. Med..

[17] Awais M., Badruddin N., Drieberg M. (2017). A hybrid approach to detect driver drowsiness utilizing physiological signals to improve system performance and wearability. Sensors.

[18] Lee H., Lee J., Shin M. (2019). Using wearable ECG/PPG sensors for driver drowsiness detection based on distinguishable pattern of recurrence plots. Electronics.

[19] Leonhardt S., Leicht L., Teichmann D. (2018). Unobtrusive vital sign monitoring in automotive environments—A review. Sensors.

[20] Chowdhury A., Shankaran R., Kavakli M., Haque M.M. (2018). Sensor Applications and physiological features in drivers’ drowsiness detection: A review. IEEE Sens. J..

[21] Choi Y., Han S.I., Kong S.H., Ko H. (2016). Driver status monitoring systems for smart vehicles using physiological sensors: A safety enhancement system from automobile manufacturers. IEEE Signal Process. Mag..

[22] Wang J., Warnecke J.M., Haghi M., Deserno T.M. (2020). Unobtrusive health monitoring in private spaces: The smart vehicle. Sensors.

[23] González-Ortega D., Díaz-Pernas F.J., Martínez-Zarzuela M., Antón-Rodríguez M. (2019). A physiological sensor-based android application synchronized with a driving simulator for driver monitoring. Sensors.

[24] Lawoyin S., Fei D.Y., Bai O. (2014). Accelerometer-based steering-wheel movement monitoring for drowsy-driving detection. Proc. Inst. Mech. Eng. Part D J. Automob. Eng..

[25] Li Z., Chen L., Peng J., Wu Y. (2017). Automatic detection of driver fatigue using driving operation information for transportation safety. Sensors.

[26] Mortazavi A., Eskandarian A., Sayed R.A. (2009). Effect of drowsiness on driving performance variables of commercial vehicle drivers. Int. J. Automot. Technol..

[27] Arefnezhad S., Samiee S., Eichberger A., Nahvi A. (2019). Driver drowsiness detection based on steering wheel data applying adaptive neuro-fuzzy feature selection. Sensors.

[28] Wilkins E., Wilson L., Wickramasinghe K., Bhatnagar P. (2017). European Cardiovascular Disease Statistics 2017.

[29] Claes N., van Laethem C., Goethals M., Goethals P., Mairesse G., Schwagten B., Nuyens D., Schrooten W., Vijgen J. (2012). Prevalence of atrial fibrillation in adults participating in a large-scale voluntary screening programme in Belgium. Acta Cardiol..

[30] Himmelreich J.C.L., Karregat E.P.M., Lucassen W.A.M., van Weert H.C.P.M., de Groot J.R., Louis Handoko M., Nijveldt R., Harskamp R.E. (2019). Diagnostic accuracy of a smartphone-operated, single-lead electrocardiography device for detection of rhythm and conduction abnormalities in primary care. Ann. Fam. Med..

[31] Haverkamp H.T., Fosse S.O., Schuster P. (2019). Accuracy and usability of single-lead ECG from smartphones—A clinical study. Indian Pacing Electrophysiol. J..

[32] Gropler M.R.F., Dalal A.S., Van Hare G.F., Silva J.N.A. (2018). Can smartphone wireless ECGs be used to accurately assess ECG intervals in pediatrics? A comparison of mobile health monitoring to standard 12-lead ECG. PLoS ONE.

[33] Duarte R., Stainthorpe A., Greenhalgh J., Richardson M., Nevitt S., Mahon J., Kotas E., Boland A., Thom H., Marshall T. (2020). Lead-I ECG for detecting atrial fibrillation in patients with an irregular pulse using single time point testing: A systematic review and economic evaluation. Health Technol. Assess..

[34] White R.D., Flaker G. (2017). Smartphone-based arrhythmia detection: Should we encourage patients to use the ECG in their pocket?. J. Atr. Fibrillation.

[35] Koltowski L., Balsam P., Glowczynska R., Peller M., Maksym J., Blicharz L., Niedziela M., Maciejewski K., Opolski G., Grabowski M. (2017). Comparison of Kardia Mobile (one lead ECGs records) with 12-lead ECGs in 100 consecutive patients with various cardiovascular disorders. Europace.

[36] Winter B.B., Winter B.B. (1983). Driven-right-leg circuit design. IEEE Trans. Biomed. Eng..

[37] Babusiak B., Borik S., Smondrk M. (2020). Two-electrode ECG for ambulatory monitoring with minimal hardware complexity. Sensors.

[38] Chi Y.M., Jung T.P., Cauwenberghs G. (2010). Dry-contact and noncontact biopotential electrodes: Methodological review. IEEE Rev. Biomed. Eng..

[39] Meziane N., Webster J.G., Attari M., Nimunkar A.J. (2013). Dry electrodes for electrocardiography. Physiol. Meas..

[40] Wood D.E., Ewins D.J., Balachandran W. (1995). Comparative analysis of power-line interference between two- or three-electrode biopotential amplifiers. Med. Biol. Eng. Comput..

[41] Acharya V. (2011). Improving Common-Mode Rejection Using the Right-Leg Drive Amplifier.

[42] Spinelli E.M., Mayosky M.A. (2005). Two-electrode biopotential measurements: Power line interference analysis. IEEE Trans. Biomed. Eng..

[43] Spinelli E.M., Pallàs-Areny R., Mayosky M.A. (2003). AC-coupled front-end for biopotential measurements. IEEE Trans. Biomed. Eng..

[44] Das S., Pal S., Mitra M. Real time heart rate detection from PPG signal in noisy environment. Proceedings of the 2016 International Conference on Intelligent Control, Power and Instrumentation, ICICPI 2016.

[45] Wei C., Sheng L., Lihua G., Yuquan C., Min P. Study on conditioning and feature extraction algorithm of photoplethysmography signal for physiological parameters detection. Proceedings of the 4th International Congress on Image and Signal Processing, CISP 2011.

[46] Huotari M., Roning J., Maatta K. Infrared and red PPG signals analysis of the healthy subjects and clinical patients. Proceedings of the Biennial Baltic Electronics Conference, BEC.

[47] Kavya R., Nayana N., Karangale K.B., Madhura H., Sheela S.J. Photoplethysmography—A modern approach and applications. Proceedings of the 2020 International Conference for Emerging Technology, INCET 2020.

[48] Yang D., Zhu J., Zhu P. SpO2 and heart rate measurement with wearable watch based on PPG. Proceedings of the 2015 IET International Conference on Biomedical Image and Signal Processing.

[49] Mohan P.M., Nagarajan V., Nisha A.A. A frame work to estimate heart rate and arterial oxygen saturation (Spo2). Proceedings of the 2017 IEEE International Conference on Communication and Signal Processing, ICCSP 2017.

[50] Longmore S.K., Lui G.Y., Naik G., Breen P.P., Jalaludin B., Gargiulo G.D. (2019). A comparison of reflective photoplethysmography for detection of heart rate, blood oxygen saturation, and respiration rate at various anatomical locations. Sensors.

[51] Postolache O.A., Silva Girão P.M.B., Sinha P., Anand A., Postolache G. (2009). Health status and air quality parameters monitoring based on mobile technology and WPAN. Int. J. Adv. Media Commun..

[52] Takahashi I., Takaishi T., Yokoyama K. (2014). Overcoming drowsiness by inducing cardiorespiratory phase synchronization. IEEE Trans. Intell. Transp. Syst..

[53] Lawoyin S., Liu X., Fei D.Y., Bai O. Detection methods for a low-cost accelerometer-based approach for driver drowsiness detection. Proceedings of the IEEE International Conference on Systems, Man and Cybernetics.

[54] Samiee S., Azadi S., Kazemi R., Nahvi A., Eichberger A. (2014). Data fusion to develop a driver drowsiness detection system with robustness to signal loss. Sensors.

[55] Aljaafreh A., Alshabatat N., Najim Al-Din M.S. Driving style recognition using fuzzy logic. Proceedings of the 2012 IEEE International Conference on Vehicular Electronics and Safety, ICVES 2012.

[56] Babušiak B., Borik S. (2016). Bluetooth communication for battery powered medical devices. J. Electr. Eng..

[57] Babusiak B., Gala M. Software tool for processing and analysis of ECG signal. Proceedings of the International Federation of Medical and Biological Engineering.

[58] Liu C., Zhang X., Zhao L., Liu F., Chen X., Yao Y., Li J. (2019). Signal quality assessment and lightweight qrs detection for wearable ECG smartvest system. IEEE Internet Things J..

[59] Hayn D., Jammerbund B., Schreier G. (2012). QRS detection based ECG quality assessment. Physiol. Meas..

[60] Kasbekar R.S., Mendelson Y. (2018). Evaluation of key design parameters for mitigating motion artefact in the mobile reflectance PPG signal to improve estimation of arterial oxygenation. Physiol. Meas..

[61] Petterson M.T., Begnoche V.L., Graybeal J.M. (2007). The effect of motion on pulse oximetry and its clinical significance. Anesth. Analg..

[62] Zhang Y., Song S., Vullings R., Biswas D., Simões-Capela N., Van Helleputte N., Van Hoof C., Groenendaal W. (2019). Motion artifact reduction for wrist-worn photoplethysmograph sensors based on different wavelengths. Sensors.

